# Laudatio to Professor Fletcher B. Taylor

**DOI:** 10.1111/j.1582-4934.2008.00367.x

**Published:** 2008-05-21

**Authors:** Florea Lupu


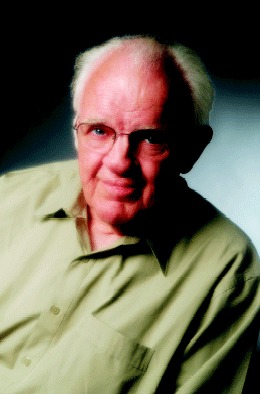


Professor Fletcher B. Taylor Jr., M.D. Member, Cardiovascular Biology Research Program Professor, Departments of Pathology, Biochemistry & Molecular Biology and Physiology & Biophysics George Lynn Cross Research Professor, University of Oklahoma Health Sciences Center OMRF Distinguished Career Scientist

For more than a half-century, cardiovascular scientist Fletcher B. Taylor, Jr., M.D., (portrayed above) has made important contributions to preclinical, translational and clinical research in vascular biology, coagulation and pathophysiology of sepsis. His work in the laboratory led to the creation of a lifesaving drug – the first and the only FDA-approved treatment for severe sepsis. So, the advent of Taylor's 80th birthday seems the ideal moment to dedicate this series of reviews on sepsis to a devoted scientist, a compassionate physician and a true Renaissance man.

Fletcher Taylor was born in San Francisco, California, in 1929. His father, an internal medicine specialist, was widely respected as a diagnostician in the East Bay area and counted among his patients the Nobel-prize winning playwright Eugene O’Neill. Although raised in an upper-class, urban atmosphere, young Taylor was drawn to the simple life. During high school, he worked as a ranch hand and cowboy, or ‘buckaroo’, as he said they were called in western Nevada. ‘I would go to high school for about six months and then spend the rest of the time up in the mountains,’ he recalls.

As a young adult, Taylor had in-depth classical music training with Dr. Alexander Rabb and Serghei Rachmaninov, aiming to become a concert pianist. These were challenging ‘coming to age’ times and he found quite hard mastering the piano as a profession. At that point, he found escape in outdoor work and spent 6 months on a farm in post-war Slovenia. Despite not pursuing it as a career, music remained very important in his life and he still plays piano with dazzling technique and intensity.

This wonderful blend of familial, cultural and educational backgrounds influenced his development of key intellectual qualities for a successful career: dedication to hard work, open-minded-ness, unlimited curiosity and a very creative imagination.

After earning his bachelor's degree in biology from Stanford University in 1952, Taylor entered doctoral studies in medicine at University of California School of Medicine in San Francisco, where he obtained his M.D. degree in 1956. During his second year of surgical internship at the Southern Pacific Hospital, his anatomy professor William O. Rinehart invited him to work on a project involving the effects of adrenalectomy on eosinophils. When a window was mistakenly left open, all the post-surgical mice in the experiment died. From what should have been a setback, Taylor instead was thrilled to observe the importance of the adrenals in protecting animals against stressful conditions, such as cold.

The root of Taylor's creativity may be traced to this failed study, for from it grew his fascination with cause–effect interactions and the ability to predict and reproduce those effects in an experimental setting. This may have contributed to his exceptional ability to read and interpret nature.

At his mentor's encouragement, he travelled to England in 1958, where he focused exclusively on research for 2 years, working with Richard Harrison (anatomy) and C.J.O.R. Morris (biochemistry) at the London Hospital. As he recalls now, ‘I sat and read for 3 months, soaking in the sights, sounds, and smells of a post-war London still choked in smog from thousands of coal fires and still recovering from six years of war followed by another eight years of rationing’.

From Harrison, Taylor learned the importance of action. He remembered, ‘After watching me do nothing for three months, Harrison said “Don't think; just do something”. His message was, don't be afraid. By all means think and plan ahead, but also do something’.

In 1959, Taylor returned to UCSF for 2 years' post-doctoral training in protein chemistry and fibrinolysis before starting his medical residency training at the same institution. During this time, he worked with Art Bickford in Julius Comroe's lab at the newly founded Cardiovascular Research Institute on the UCSF campus and in 1963 published in *Nature* a paper on the role of platelets in the dissolution of blood clots [1]. This was when Taylor started his first animal model studies following the *in vivo* effects of infusion of the plasminogen activator streptokinase on the cardiovascular system in dogs [2].

In parallel, as a resident physician, he applied his research experience in protein chemistry to studies and treatment of patients with severe sepsis who came under his care. This proved to be a turning point in his career, and since that time, Taylor's work became focused on methods for identification of the patho-physiology of severe sepsis and using the information to treat this disorder.

First, he believed that the activity of the coagulation enzymes was overwhelmed by the fibrinolytic enzymes that dissolve the clots. For this, he first assembled a research group at the University of Pennsylvania, where he acted as the Head of allergy and Immunology. Here he focused on the structure and function of a bacterial protein called streptokinase that can convert the pro-enzyme plasminogen into the potent clot-dissolving enzyme plasmin [3]. Also, he adapted a dilute whole-blood clot lysis assay as a screening method for patients with thrombotic complications [4] and made seminal observations on the interplay of coagulation and complement activation in sepsis and the role of platelets and complement in fibrinolysis. These projects were conducted in collaboration with Dr. Hans Muller-Eberhard [5] and Peter Ward [6], two major players in the field of complementology, and resulted in Taylor's election to the American Society of Clinical Investigation.

In 1974, Taylor moved to the University of Oklahoma to lead a new program in experimental pathology. Then, early in 1982, he moved to the Oklahoma Medical Research Foundation (OMRF), where he established a research group in thrombosis that further expanded as today's Cardiovascular Biology Research Program. He remains an active member in the program today and was named an OMRF Distinguished Career Scientist in 2006.

During this time at OMRF, Taylor started his collaboration with Chuck and Naomi Esmon, expanding his work to include not only the blood but also the endothelial lining of the vessel wall [7]. This highlighted the importance of the vasculature as a functional unit during the acute response to bacterial infection, or severe sepsis.

With his unrestrained enthusiasm to learn new things, Taylor progressed from test-tube assays to animal models. While working with Dr. Hinshaw on arterial-venous by-pass experiments using a pump system in dogs exposed to endotoxemia, they noticed that the procedure led to an unexpected protection against endotoxin-induced shock. At that time, based on the almost contemporaneous observations of Esmon and Owen [8], they postulated that the blood pumping generated sufficient thrombin to turn the endogenous protein C (PC) into activated PC (APC), but not enough to cause blood clotting. This hypothesis was tested directly by infusing thrombin into dogs challenged with endotoxin. Surprisingly, the thrombin infusion protected the dogs from death. This landmark observation led to studies using APC in the baboon model of *E. coli* sepsis, that ultimately led to the development of APC as the first proven treatment for adult severe sepsis of patients who are at high risk of death [7]. The drug, which was produced and commercialized by Eli Lilly and Company as Xigris®, was approved by the Food and Drug Administration in 2001.

During the last 15 years, Taylor's work has focused on understanding the pathophysiology of sepsis, especially on the stages of the disease and the mechanisms that control its progression. Having spent many years caring for patients, Taylor sought to understand why some patients treated with Xigris® survive a sepsis episode while others die. He began to realize that the lethal baboon model of sepsis he developed with Lerner Hinshaw and used in the rescue APC experiments [7] was driven not only by the pathogen [9] but also by other pathophysiological processes.

Working with critical care physician Gary Kinasewitz [10] and later with Florea Lupu [11–13], a vascular biologist, Taylor learned that exposure of the baboons to lower *E. coli* doses could also kill some animals, but much slower and through a different patho-physiology. The host response to sublethal doses of bacteria exhibited at least two stages, each driven by unique pathophysio-logic processes. The second stage is driven by ischaemia-reperfusion injury initiated during the first stage and can develop into progressive and frequently lethal organ failure. The results obtained in the animal models were further translated to the clinical setting by Kinasewitz *et al*. [14].

Taylor made major contributions to basic science and clinical literature, authoring or co-authoring over 150 scientific articles, reviews and book chapters. He has received multiple honors and prizes, including the Cochems Prize for Outstanding Research in Cardiovascular/Thromboembolic Disease (1968), and held numerous distinguished positions, such as George Lynn Cross Research Professor at the University of Oklahoma (1979), the H.A. and Mary K. Chapman Chair in Medical Research at OMRF (1985), and OMRF Distinguished Career Scientist (2006).

He also has served on multiple advisory boards and study sections of funding organizations, such as NIH, NASA, the American Heart Association and American Red Cross, and as reviewer for many medical and biological journals, including *Blood, Journal of Clinical Investigation, Shockand Critical Care Medicine*.

In addition to his distinguished accomplishments in research, Taylor has maintained contact with patients as an attending physician at Veteran's Administration Medical Center and University Hospital in Oklahoma City.

Taylor's collaborative spirit has brought him to partner with countless other scientists during his career, and he considers those partnerships the key to his research success. His close collaborator for many years, Chuck Esmon, feels that Taylor's unique personality ‘made it possible to maintain effective collaborative interactions over many years without personal pride disrupting productive interactions, as all too commonly occurs in the competitive scientific arena’.

Besides being a distinguished cardiovascular physiologist and clinician, Dr. Taylor was, and continues to be, an exceptional mentor whose remarkable intellect and biomedical knowledge has influenced the careers of many scientists, including the author of this tribute.

As the founder of the Cardiovascular Biology Research Program at OMRF, he takes pride in his outstanding trainees and collaborators he has recruited over the years, among them Chuck and Naomi Esmon, Rodger McEver, Jim Morrissey and many others who now are successful scientists and hold leadership positions in the department or moved to other prestigious institutions. In his always-humble way, Taylor says, ‘I always want to be surrounded by people more clever than myself’.

Fletcher Taylor has a contagious curiosity and keen interest in new ideas and approaches. What I found extremely encouraging for me, as a newcomer to the sepsis field, was his great enthusiasm and the interest with which he embraced the work of others, including mine, always showing appreciation for what looks promising and for the hard work that went into it, rather than pointing out the mistakes.

With his positive outlook on life and towards others, Taylor continues to promote a spirit of camaraderie and fosters a mutually supportive environment. And his appreciation for those ‘in the trenches’, the technical staff, has gained him a group of very dedicated and loyal coworkers, some of whom have remained with him for over three decades. In fact, rather than spending the money he received from the APC patent on himself, he used it to endow the Al Chang Chair in Cardiovascular Biology at OMRF, named for Taylor's friend and technical assistant of more than 25 years, who passed away in 2004.

Besides supporting his people to achieve their maximum potential, Taylor always has taken very seriously the responsibility to promote his research field. He constantly pushes for knowledge to be translated into practice, or paraphrasing Goethe, ‘knowing is not enough, we must apply’.

Today, even as he nears 80 years of age, Taylor spends 3 weeks of every month working the land on his farm in Macclesfield, North Carolina. But he still dedicates 1 week each month to OMRF, advising, encouraging and sharing ideas. This compassionate thinker, scientist and physician continues to impress those around him with his broad knowledge and keen insight, his exceptional ability to integrate basic physiologic principles with clinical parameters, and his innate gift for separating the trivial from that which has genuine significance.
